# Early Life Stress and Childhood Aggression: Mediating and Moderating Effects of Child Callousness and Stress Reactivity

**DOI:** 10.1007/s10578-018-0785-9

**Published:** 2018-02-13

**Authors:** Dominika A. Winiarski, Melissa L. Engel, Niranjan S. Karnik, Patricia A. Brennan

**Affiliations:** 1 Department of Psychiatry, Rush University Medical Center, 1700 W. Van Buren St., Suite 5827A, Chicago, IL 60612 USA; 2 Institute of Child Development, University of Minnesota, Minneapolis, MN USA; 3 Department of Psychology, Emory University, Atlanta, GA USA

**Keywords:** Aggression, Callousness, Cortisol reactivity, Stress

## Abstract

Early life stress (ELS) has been implicated in the development of aggression, though the exact mechanisms remain unknown. This study tested associations between ELS, callousness, and stress reactivity in the prediction of school-age and persistent early childhood aggression. A longitudinal sample of 185 mother–child dyads completed a lab visit and mothers completed an online follow-up when children were preschool-aged and school-aged, respectively. Physiological and behavioral measures of stress reactivity were collected during the preschool period. Ratings of child aggressive behavior, ELS, and callousness were collected as well. The results suggested that ELS was related to measures of both school-age and persistent early childhood aggression, and that callousness had a mediating role in this process. Cortisol reactivity also moderated the association between ELS and persistent childhood aggression, such that the ELS–aggression relationship was stronger among children who had higher levels of cortisol reactivity during the preschool period. Clinical implications are discussed.

## Introduction

Early life stress (ELS) has been implicated in a variety of emotional and behavioral problems, one of which is aggression. Although there is considerable evidence linking ELS to adverse behavioral outcomes in adolescence and later adulthood, much less is known about how ELS is implicated in the development of behavioral and physiological regulation in childhood, and how that in turn relates to higher levels of aggression in school age. The present study aims to address this developmental gap in the literature.

### Linking the HPA Axis with Early Life Stress and Aggression

Individuals with histories of ELS (e.g., physical and emotional neglect, abuse, loss of a parent) often show problems with aggression well into adulthood [1, 2]. Recent research has also shown that ELS is related to the development of aggression among school-aged children [3]. Nevertheless, although the link between ELS and aggression has been established, the specific processes by which ELS contributes to higher rates of aggression remain less clear. One proposed pathway by which ELS may affect behavior is through changes in the hypothalamic–pituitary–adrenal axis (HPA [4]), which is the primary pathway for the mammalian stress response. The end-result of this pathway is the release of the glucocorticoid cortisol. Previous research has suggested that dysregulation of the HPA axis is involved in the pathogenesis of aggressive behavior [5] and mood disorders, as well as later adolescent delinquency [6]. The timing of stress exposure can be especially important for the developing HPA axis, and can set the stage for future outcomes [7].

The detrimental effects of ELS on the developing HPA axis (and subsequent dysregulation of emotions and behavior) have been well elucidated in both the human and animal literatures [8, 9]. The adverse physical and psychological consequences of prolonged cortisol elevations have similarly been illustrated in the literature [10, 11]. Moreover, previous research suggests that ELS is linked to problems with emotion regulation among children who also demonstrate disrupted HPA axis functioning [12], suggesting that the HPA axis may play a critical role in shaping maladaptive behaviors stemming from ELS. Cortisol reactivity may act as a mediator, such that a child exposed to early life adversity develops HPA axis dysregulation, which in turn makes him more likely to act out aggressively. Alternatively, cortisol reactivity may act as a moderator between ELS and aggression, with children exposed to ELS being more likely to evidence aggression, if they were also physiologically more vulnerable (via cortisol reactivity) to this behavioral outcome.

Research findings linking HPA axis dysregulation to aggression among older children and adolescents are conflicting in terms of direction of effect. Some studies of adolescents and school-age children have found that aggression negatively correlates with cortisol levels [13], whereas others have found that this relationship can be better explained by high levels of callous–unemotional (CU) traits rather than antisocial behavior in general [14, 15]. Given the empirically established relationships between ELS and the developing stress reactivity system and aggressive behavior, it is important to further explore the role that cortisol plays in linking ELS to aggressive behavior. Given the lack of consensus in the literature about the exact processes by which cortisol has an effect on the development of aggression, the current study will examine HPA axis reactivity as both a potential mediator and moderator in the relationship between ELS and childhood aggression.

### Behavioral Indicators of Stress Reactivity and Aggression

Behavioral indicators of stress reactivity have also been linked to later aggression. Children with high reactivity and poor behavioral inhibition tend to be at higher risk of future behavioral problems [16, 17]. Several prospective studies link aggressive behavior and early childhood temperament, especially negative emotionality and intense reactive responding, with continued aggression across the lifespan [18–20]. Similarly, Gartstein et al. [21] found that children with high levels of negative emotionality (i.e., sadness and frustration) measured throughout infancy and toddlerhood exhibited higher levels of externalizing behavior problems at age five.

Since emotion regulation is a key component in the development of appropriate regulatory social behaviors, one can see how poor stress reactivity in childhood may be linked to the later development of aggression [22, 23]. Individuals with no regulatory skills in place are much more sensitive to the effects of negative affect brought on by interactions with a stressful environmental context, and may be more likely to act out aggressively [24]. Because of the link between poor stress reactivity and deficits in emotion regulatory abilities, this study conceptualizes measures of preschool stress reactivity as measures of a child’s emerging abilities to effectively regulate emotion. Moreover, given the previously established findings that ELS can affect stress reactivity at both a physiological and behavioral level, the present study will evaluate multiple proxies of stress reactivity as mediators and moderators of the ELS–aggression relationship.

Furthermore, the current study may inform well-established theories of criminal offending, such as Agnew’s general strain theory of crime. Briefly, Agnew postulates that criminal behavior is modulated by motivations and constraints, and is more likely to occur when an individual feels “pressured” by negative affective states such as anger [25]. Agnew has suggested that biological factors and individual differences may contribute to interpersonal variations in crime [26]. A better understanding of these interpersonal variations (i.e., emotion regulation) may help inform sociological and psychological frameworks of crime, and could reduce the societal and psychological burdens of persistent criminal trajectories.

### The Role of Callousness in Aggression

CU traits are typically characterized by a lack of guilt and remorse, superficial expression of emotions, and a lack of concern for the feelings of others [27–29]. Previous studies demonstrate links between many constructs of CU traits, such as poor fear conditioning and fearlessness in preschool-aged children and behavioral outcomes in late childhood and adulthood [30, 31]. Considerable evidence also suggests that, among children at risk for clinically significant aggressive behavior, there may be a particularly treatment-resistant and at-risk group of youth who show high levels of aggression, high levels of CU traits, and low levels of physiological arousal [32]. To the authors’ knowledge, no studies to date have examined CU traits as mediators between ELS and childhood aggression, but research linking childhood abuse to psychopathy in adults [33] suggests that ELS may predict early CU traits, which in turn predict to later aggression. Therefore, callousness will be examined as both a mediator and a moderator of the ELS–childhood aggression relationship. We generally expect ELS to predict higher aggression, but we also anticipate that CU traits in childhood may moderate this association.

### The Role of Maternal Mental Health

The relationship between maternal mental health and adverse child behavioral, social, and emotional outcomes has been well-established in the literature [34, 35]. For example, Holmes [36] found that poor maternal mental health was associated with aggressive behavior in children, and that maternal mental health mediated the relationship between intimate partner violence and aggression. Specifically, while intimate partner violence did not have any direct effects on aggression, it seems that maternal mental health plays a key role. Maternal mental health problems were also associated with lower maternal warmth, which was then directly related to aggressive behavior problems. Others have similarly found that maternal anxiety and somatization are related to higher levels of mother-reported aggressive behavior on the Child Behavior Checklist [37]. Recurrent maternal major depressive disorder has also been associated with higher levels of chronic and episodic social stress in school-age and early adolescent-aged children [38]. The present study will build off this established link in the literature to evaluate how ELS relates to aggression via stress reactivity in a sample of children exposed to high levels of maternal mental health problems.

### Current Study

Although previous studies have established links between aggression in school-aged children and later adolescent delinquency [39], as well as future adult criminality [40], there is less research identifying the various early childhood physiological and behavioral risk factors that predict both school-age and early childhood persistent aggressive behavior. The proposed study will add to what is known about the development of aggression by examining associations between ELS, preschool markers of stress reactivity, and childhood aggression. Prospectively collected preschool data on child aggression, child behavioral responses to a lab-based frustration task, and child cortisol measures were supplemented by follow-up school-age measures of parent and alternate caregiver ratings of child aggression and CU traits.

We hypothesized that higher levels of ELS would predict to higher levels of school age aggression and persistent child aggression. We also hypothesized that high levels of ELS would predict to higher behavioral and physiological stress reactivity in preschool age as well as higher callousness in childhood, which would in turn relate to higher levels of caregiver-reported school-age aggression as well as higher levels of early childhood persistent aggression. Finally, we predicted that higher levels of behavioral and physiological stress reactivity as well as higher levels of mother-reported callousness would moderate the relationship between ELS and both persistent and school-age aggression, such that children with ELS and high levels of callousness would be more likely to demonstrate aggression than the other children in our sample.

## Method

Participants were drawn from an existing sample of 219 mother–child dyads, 178 of whom were recruited from the Emory Women’s Mental Health Program (WMHP) within the Emory University School of Medicine Department of Psychiatry. The remaining 41 women in the sample were recruited from the community at the time of the preschool study visit, and did not take psychotropic medications during pregnancy, as verified by obstetrical records. Participants recruited from the WMHP and control participants did not differ on any demographics relevant to the current study (e.g., mother age, child age, mother/child ethnicity) except for number of children in the home (*p* = 0.046) with control participants having more children in the household than participants recruited from WMHP.

This study was approved by the Institutional Review Board of Emory University. Mothers and alternate caregivers provided consent for their participation in both phases of the study, and were financially compensated at each time point. Mothers also provided consent for their children’s participation in the preschool study, and children received a small toy as compensation.

### Demographics of the Sample

During the preschool phase of the study, children’s ages ranged from 2.5 to 5.5 years (*M* = 3.7, *SD* = 0.89) and mother’s ages ranged from 21 to 49 years (*M* = 36.9, *SD* = 5.0). Child sex was evenly split (*N* = 110 females). The women in the sample were predominantly White (82.6%), although other ethnicities were represented as well (9.6% African American, 3.2% Hispanic, 2.3% Asian, 1.4% Biracial). The children in this sample also represented a variety of racial and ethnic groups (79% White, 9% African American, 2% Latino, and 10% biracial or other). Mothers were well-educated (6.8% graduated 2-year college, 32.9% graduated 4-year college, 40.2% completed graduate/professional school), and most were married (81.7%). A total of 83.9% of mothers in the sample were diagnosed with one or more DSM-IV-TR Axis I diagnoses across their lifetimes. Furthermore, 59.4% of the mothers were undergoing mental health treatment (e.g., individual therapy, psychiatric services) at the time of the preschool visit.

The mother–child dyads that were followed up at school-age (*N* = 185) represent approximately 85% of the participants who were initially recruited for the preschool phase of the study. Participants lost to follow-up had significantly lower levels of maternal education compared to those retained [*t*(*df* = 215) = − 2.45, *p* = 0.015], with 86.9% of mothers having a Bachelor’s degree or higher. During the school-age phase of the study, children’s ages ranged from 5 to 10 years (*M* = 7.16, *SD* = 1.20) and mother’s ages ranged from 24 to 53 years (*M* = 40.85, *SD* = 4.87). Child sex was again evenly split (51.4% male, 48.6% female). Mother’s employment status varied (28.7% reported being unemployed/not working, 24.9% were employed part-time, 44.3% were employed full-time, and 1.1% were retired).

### Procedure

During the preschool phase of the study, participants completed several measures of behavioral, cognitive, and language development during a lab visit. Mothers also completed questionnaires about their current symptoms of depression and anxiety, as well as stressors they experienced during their child’s lifetime. Children’s aggressive behavior was rated by an alternate caregiver (e.g., grandmother, father, babysitter, etc.) to supplement our maternal-report measures. During this visit, children also participated in a lab-based frustration task and provided two saliva samples.

In the follow-up school-age study, data were collected via a secure online database called REDCap. Permission to re-contact, along with contact information, was obtained during the preschool phase of the study. Mothers received a direct hyperlink to the online measures. Like the preschool phase of the study, behavioral questionnaires were also completed by an alternate caregiver using the same REDCap database.

#### Early Life Stress

To assess ELS, mothers were asked to complete a 57-item self-report measure called the Life Experiences Survey [41] during the preschool lab visit, endorsing the specific number of events they had experienced in the past 6 months or since their pregnancy with the enrolled child (i.e., child’s “lifetime”), as well as subjectively evaluating how positively or negatively those events impacted them. The kinds of stressful life events represent both proximal (e.g., experiences directly affecting the child) and distal (i.e., experiences affecting the family environment) factors [42]. The lifetime subjective ratings of ELS were used in all analyses to capture the impact of cumulative stress exposure across the child’s life.

#### Behavioral Stress Reactivity

Two batteries of the preschool version Laboratory Temperament Assessment Battery (Lab-TAB), the “Attractive Toy in a Transparent Box” and “Impossibly Perfect Green Circle” [43] were used to measure behavioral stress reactivity in the preschoolers. In the first task, children were asked to pick one of two toys (ball or slinky) that was then placed inside a box and locked by the one of the research assistants. The assistant then instructed the child to find the correct key on a set of keys and to open the box with that key. Before leaving the room, the experimenter told the child, “most kids do this fast.” The child was left alone for 2 min. After 2 min, the research assistant entered the room and apologized to the child, saying that it was her fault that the child could not open the box because she had the correct key all along. She then helped the child open the box and play with the toy.

In the second frustration task, “Impossibly Perfect Green Circle,” the research assistant asked the child to draw by free-hand “a perfect green circle.” After each attempt, the child was given negative feedback (e.g., “that’s too pointy,” “that has an edge”) regardless of how well the circles were drawn. After two attempts, the research assistant showed the child two perfectly traced circles and said, “the last two kids that were here drew these circles. These circles are perfect. They thought this was easy.” The child was asked to continue drawing circles for 3 min. At the conclusion of the task, the research assistant selected one of the circles that the child drew, and told the child that it was indeed a good circle.

Both Lab-TAB tasks were recorded for later offline behavioral coding. First, each video was divided into 10-sec epochs. Then for each epoch, a trained researcher coded each child along the following dimensions: intensity of anger expression, presence of bodily anger, peak intensity of frustration, intensity of sadness expression, presence of bodily sadness, and peak intensity of gaze aversion. Reliability coding was conducted by another trained coder on 10% of the videos. Reliability was high (α > 0.8) across all behavioral codes. separate principal components analyses (PCA) were conducted on the different behavioral variables of the clear box (CB) task and green circles (GC) task. Latent variables were extracted using principal axis factoring and oblique rotation [44]. Two factors, anger and sadness, were identified using the eigenvalue rule [45], which recommends including elements with values > 1, and verified through scree plot analysis [46]. Anger expression (0.736) and bodily anger (0.769) loaded onto the anger factor for each task, which was subsequently used in all statistical analyses of behavioral reactivity.

#### Physiological Stress Reactivity

Salivary cortisol samples were collected from the children during the preschool study lab visit at two time points: after they arrived at and acclimated to the lab (baseline) and 20 min after the completion of the frustration tasks. Study times were standardized to control for diurnal variations in cortisol. All participants were instructed to not eat the morning of the lab visit. To aid in sample collection, children were asked to chew on a piece of cotton dipped in a very small quantity of Kool-Aid™. Previous studies have suggested that, when used consistently and in small quantities [47], such noninvasive saliva “stimulant” collection methods can be useful, should not distort either within or between subject comparisons, and can yield reliable measures of cortisol [48–51]. Furthermore, given the young age of the subjects (2.5–5 years), this method was deemed most appropriate in increasing compliance.

After chewing the cotton for 1 min, a research assistant retrieved the cotton from the child, put it inside the lumen of a 0.5 ml syringe, and squeezed out about 1 cc of saliva into a small tube. All saliva samples were frozen and stored at − 20 °C before being transported for assay to the Yerkes National Primate Research Lab at Emory. Samples were then thawed, vortexed, and centrifuged to remove any particulate matter. Salivary cortisol was assayed using an enzyme immunoassay kit (Diagnostic Systems Laboratories; DSL, Webster, TX), catalogue number DSL-10-67100. This assay procedure has an analytical sensitivity of 0.10 mg/dl, using 25 ml of saliva. The intra- and inter-assay coefficient of variation is 4.1 and 7.2%, respectively. Each sample was assayed in duplicate, and duplicate tests with an error of > 20% were retested. Duplicate test results were averaged and this value for cortisol was used in analyses.

Cortisol values were heavily skewed at time 1 (initial collection at the start of the lab visit; 6.79) and at time 2 (post-frustration task; 7.40). Because none of the obtained cortisol values were biologically implausible, and they all fit the standard curve of the assay, extreme values were Winsorized to retain participant data in the analyses. To capture cortisol reactivity, time 1 values were regressed on time 2 cortisol values, thereby creating a residualized change score that was used in all subsequent analyses. Previous studies have advocated using this metric as a more statistically reliable measure of cortisol reactivity than calculating a (pre- and post-stressor task) difference score [52].

#### Aggression

To assess levels of aggression in preschool children, each child’s mother and an alternate caregiver were asked to complete the 100-item Preschool-Age Child Behavior Checklist (CBCL [53]) which evaluates children’s behavior across several domains of functioning.

Aggressive behaviors in the school-age follow-up were measured using the School-Age CBCL [54], which is very similar to the preschool-age form. Again, both mothers and alternate caregivers completed this form. The aggressive behavior subscale was used as the outcome variable in analyses. Examples of items that load onto this subscale include “gets in many fights,” “hits others,” and “physically attacks people.” Reliability was high (α > 0.8) across all measures. Given the modest correlations between maternal and alternate-caregiver rated aggression during the preschool assessment (*r* = 0.463, *p* < 0.01) and school-age follow-up (*r* = 0.583, *p* < 0.01), the researchers combined mother and alternate caregiver ratings of children’s aggression into overall standardized preschool aggression and school-age aggression variables. In addition, the researchers created a persistent aggression variable which identified the children whose aggression was rated as above the mean on the combined mother and alternative caregiver measure of aggression at both assessment time points (*N* = 42).

#### Callous–Unemotional Traits

For the school-age follow-up study, mothers completed the Inventory of Callous–Unemotional Traits (ICU [55]). They were asked to read a series of 24 statements, and rate their children on a scale of 0 (“not true at all”) to 4 (“definitely true”). Items include: “expresses his/her feelings openly”; “seems very cold and uncaring”; “shows no remorse when he/she has done something wrong.” Three factors emerge on the ICU (callousness, uncaring, unemotional) [55]. For the purposes of these analyses, the focus was on the callousness factor. Reliability for this scale was moderate (α = 0.663). Given that there are no widely-accepted clinical cut-offs for the ICU [56, 57], callousness was entered as a continuous variable in all moderation analyses.

## Statistical Approach

Linear and bivariate logistic regression analyses were utilized to assess the associations between lifetime ELS measures and school age/persistent child aggression measures. Separate linear and bivariate logistic regression analyses were then undertaken to examine the potential moderating role of child behavioral and cortisol reactivity, as well as callousness in these ELS–aggression relationships. Variables were mean centered before being entered as predictors in the moderator analyses.

A series of PROCESS models [58] were then utilized to test the hypotheses concerning mediation. In these analyses, simple mediation models (model 4) were used to examine the pathway from ELS to school age aggression/persistent childhood aggression through measures of preschool stress reactivity (CB/GC anger and cortisol reactivity) and child callousness. PROCESS uses an ordinary least squares or logistic regression-based path analytic framework to estimate direct and indirect effects, and produces bootstrap standard errors and 95% confidence intervals for the specific indirect effects using 5000 bootstrap samples. The absence of a zero value within the bootstrap confidence interval is suggestive of an indirect effect.

## Results

### Descriptive Analyses

Correlations between ELS, aggression, emotion regulation, and moderating variables are presented in Table 1. All aggression measures were significantly and positively related to one another. Callousness significantly and positively related to each aggression measure, but not to either of the behavioral indices of stress reactivity or to cortisol. Child sex did not relate to any stress reactivity or aggression variable. With the exception of GC anger (small, negative correlation with callousness) physiological and behavioral measures of reactivity were not correlated with one another or with child measures of callousness.

**Table 1:** Intercorrelations for study variables

Variable	1	2	3	4	5	6	7	8	9
1. Cortisol	–	− .019	− .008	.033	− .011	.027	.073	− .009	− .085
2. CB anger		–	.150	.102	.015	.068	− .124	.074	.057
3. GC anger			–	.280**	.185*	.228**	− .113	− .168*	− .030
4. Preschool-age aggression				–	.500**	.669**	− .164*	.350**	− .007
5. School-age aggression					–	.745**	− .103	.439**	.021
6. Persistent aggression						–	− .143	.367**	− .015
7. LES lifetime subjective rating							–	− .188	− .006
8. Callousness								–	.002
9. Child sex									–

*p < 0.05; **p < 0.01

### Determining Covariates

During the preschool visit, mothers completed a 20-item Child Health Questionnaire which was designed to address several variables that have been shown in the literature to influence cortisol [59–62]. None of the health variables were significantly related to our measure of physiological reactivity.

Demographic variables previously associated with childhood aggression and CU traits were tested as potential covariates. Although there was not a standardized measure of socioeconomic status (SES) available in the present study, maternal education, which previous studies have shown to be related to SES [63], was tested as a potential covariate. Child sex was also tested as a possible covariate. Furthermore, because many of the women in this sample were recruited from a treatment center, maternal psychiatric history as assessed by the Structured Clinical Interview for DSM-IV Axis I Disorders [64] was also assessed as a potential covariate. No demographic or maternal mental health covariates were identified for the preschool, school age, and persistent aggression variables. There was no multicollinearity between moderators and independent variables.

### Hypothesis Testing

#### Mediation Analyses

Our mediation hypothesis was that high levels of ELS would predict to higher callousness, as well as behavioral and physiological stress reactivity, which would in turn relate to higher levels of aggression in school-age as well as higher levels of early childhood persistent aggression. Results for this hypothesis for both school-age aggression and persistent early childhood aggression outcomes are presented in Table 2. PROCESS models revealed significant indirect effects of callousness and behavioral stress reactivity (anger in response to green circles stress task) in the relationship between lifetime ratings of ELS and school age aggression. Specifically, more negative life events predicted higher levels of child callousness and behavioral anger, which in turn predicted higher levels of school age aggression. In addition, callousness had a significant indirect effect on the relationship between lifetime ratings of ELS and persistent aggression, such that higher lifetime stress predicted higher callousness, which in turn predicted persistent aggression.

**Table 2:** Summary of mediation analyses

	Coefficient (SE)	LLCI	ULCI
School-age aggression			
Lifetime ELS			
Cortisol	0.0001 (0.0010)	− 0.0016	0.0028
GC anger	− **0.0027 (0.0019)**	− **0.0080**	− **0.0001**
CB anger	0.0010 (0.0014)	− 0.0006	0.0053
Callousness	− **0.0083 (0.0036)**	− **0.0173**	− **0.0026**
Persistent aggression			
Lifetime ELS			
Cortisol	0.0004 (0.0042)	− 0.0055	0.0112
GC anger	− 0.0068 (0.0050)	− 0.0196	0.0005
CB anger	0.0007 (0.0039)	− 0.0046	0.0121
Callousness	− **0.0215 (0.0117)**	− **0.0490**	− **0.0048**

Bolded values indicate significant findings

*GC* green circles frustration task, *CB* clear box frustration task

#### Moderation Analyses

We also examined callousness, behavioral stress reactivity and cortisol reactivity as potential moderators between ratings of ELS and school-age and persistent aggression. As shown in Table 3, only cortisol reactivity was found to moderate the relationship between lifetime ratings of ELS and child persistent aggression. Post-hoc logistic regression analyses revealed that lifetime ratings of ELS were related to persistent aggression for children with higher levels of preschool cortisol reactivity [Wald = 6.290, *p* = 0.012, Exp(B) = 0.789], but not children with lower levels of cortisol reactivity [Wald = 0.196, *p* = 0.658, Exp(B) = 1.025].

**Table 3:** Summary of moderation analyses

Lifetime ELS → school-age aggression			
	Change *R*^2^	*F*	*p*
Cortisol	.012	2.609	0.108
GC anger	.006	1.096	0.297
CB anger	.010	1.582	0.210
Callousness	.001	0.206	0.651

Lifetime ELS → persistent aggression			
	Coefficient (SE)	Wald	*p*
Cortisol	**− 0.078 (0.038)**	**4.279**	**0.039**
GC anger	0.022 (0.027)	0.658	0.417
CB anger	0.041 (0.028)	2.176	0.140
Callousness	− 0.002 (0.009)	0.038	0.846

Bolded values indicate significant findings

*GC* green circles frustration task, *CB* clear box frustration task

## Discussion

This study contributes to the aggression literature by demonstrating several possible routes through which ELS predicts to aggression among young children. Results suggest that maternal ratings of ELS experienced between pregnancy and the preschool phase of development has only indirect and interactive influences, rather than direct influences on later childhood aggression.

Several notable findings emerged from the present analyses. The first was the important role that callousness played in the relationship between stress and aggression. More negative lifetime events were related to both higher levels of behavioral measures of anger (GC, specifically) and higher parent-rated callousness, which in turn predicted to more aggression displayed during school-age. It is likely that the GC task elicited more frustration in children than the CB task given the nature of the repetitive negative feedback of this task. Similarly, higher levels of lifetime stress also predicted to callousness, which in turn predicted to persistent aggression across childhood. These intriguing findings are consistent with Frick and White, who argued that CU traits are particularly important for distinguishing subgroups of antisocial youth, and that these traits predict a more stable form of aggression that begins earlier in life [29]. A better understanding of how early physiological and behavioral markers of stress reactivity can put children at risk of developing later disruptive behaviors can inform early intervention efforts aimed at reducing persistent aggression. Although further longitudinal follow-up with the present sample is needed to determine whether this is a stable predictor of continued problems into adolescence, these results certainly demonstrate that callousness is a useful variable to assess for when evaluating behavioral outcomes among children exposed to high levels of psychosocial stress.

Another important finding that emerged was that cortisol reactivity moderated the relationship between lifetime ratings of ELS and persistent childhood aggression. Specifically, lifetime stress was related to persistent aggression only for children with higher levels of cortisol reactivity. Although lower cortisol levels, thought to reflect callousness, are generally linked to adolescent and adult aggressive and criminal behavior, Alink et al. [65] found that externalizing behavior is generally associated with *higher* basal cortisol in preschool-aged children and with lower levels during the school-age period. It is possible that, among children who are developing in high-stress environments, the hyper-responsive HPA axis downregulates between early and middle childhood. Of course, additional follow-up with this sample would be needed to test this intriguing hypothesis.

There are several strengths of the present study, including the longitudinal nature of the design, as well as multiple raters of child behavior and stress reactivity. There was a high rate of participant follow-up from the preschool to the school-age period, and only minor differences between those who participated in the follow-up study and those who did not. Also important is the fact that this study examined the development of aggression among an understudied sample of children in the aggression literature (that is, a higher SES sample). Although from primarily higher SES backgrounds, these children are expected to be exposed to higher rates of stress given parental psychopathology.

Even though this study examined several markers of stress reactivity, and while the behavioral indicators were correlated in the expected direction, neither one of the behavioral indicators was correlated with cortisol. As such, these measures were conceptualized as separate proxy measures of stress reactivity. This distinction between physiological and behavioral indicators of emotion reactivity is consistent with the work of researchers who argue that response coherence among behavioral and physiological indices is not necessary [66]. This lack of response coherence suggests that additional research is needed to further interrogate the best ways to conceptualize and measure emotion regulation, particularly among younger samples of children.

### Potential Limitations and Future Directions

These findings should be interpreted in the context of some notable limitations. As mentioned above, the sample of mothers and children in this study were of higher SES and had access to mental health services. Less is known about the risk factors for the development of aggression among higher SES samples, but it is possible that the effects of ELS on emotion regulation were buffered by mothers’ mental health treatment. In other words, mothers may be learning strategies to manage their own mental health problems, thus reducing the child’s exposure to mental health-related stress. Likewise, mothers may also be modeling appropriate stress reduction strategies to their children which may help curb some early aggression. Thus, future studies should explore the link between ELS, emotion regulation, and aggression using a sample of patients with diverse socioeconomic and mental health treatment profiles.

The current study did not distinguish between proactive and reactive subtypes [67, 68], nor did it distinguish between physical and relational aggression. Future studies should more carefully distinguish between the different *types* of aggressive behavior. It is also important to consider other potential mechanisms for the association between ELS and childhood aggression, including parenting and peer group affiliation, which will become especially salient if this sample is followed up through middle childhood and into adolescence.

Our findings may also have been limited by the sole use of maternal report concerning ELS. Future studies of this type should include both objective and subjective measures of stress from not only the mothers, but from their children as well. This would allow for an examination of how children’s perceptions of stress interact with their reactivity to predict to outcomes.

## Conclusions

This multi-method study explored the mediating and moderating effects of physiological and behavioral stress reactivity and callousness on the relationship between ELS and school-age and persistent childhood aggression (i.e., preschool-age to school-age). By exploring variables that play an important role in the emergence and maintenance of aggressive behaviors, these findings contribute to the emerging literature within the field of developmental psychopathology on the early indicators of clinically significant disruptive behavior problems. The results of the present study further reinforce the notion that researchers should specifically evaluate levels of callousness among young children who are identified as at-risk for aggressive behavioral problems. Therefore, these findings should be considered as new iterations of interventions are developed for treating disruptive behaviors in youth.

## Summary

This longitudinal study tested associations between ELS, callousness, and stress reactivity in the prediction of school-age and persistent early childhood aggression. The researchers used a multi-method approach to evaluate children during preschool-age and again during a school-age follow-up. Examiner ratings of child behavior in response to stress, parent and alternate-caregiver behavioral reports, and a physiological indicator of stress reactivity (i.e., cortisol) were used to evaluate the variables of interest in the present study. In summary, the authors found that ELS was related to school-age aggression among children with high levels of mother-reported callousness and more behavioral reactivity to a frustration task. Furthermore, ELS was also related to persistent aggression among children with higher levels of callousness. Cortisol reactivity moderated the relationship between ELS and persistent aggression, such that lifetime ratings of ELS were related to persistent aggression only for children with higher levels of preschool cortisol reactivity. These results may inform the development of future interventions for aggressive youth, as well as prevention efforts for at-risk children.
