# Correction to: Early Life Stress and Childhood Aggression: Mediating and Moderating Effects of Child Callousness and Stress Reactivity

**DOI:** 10.1007/s10578-018-0796-6

**Published:** 2018-02-24

**Authors:** Dominika A. Winiarski, Melissa L. Engel, Niranjan S. Karnik, Patricia A. Brennan

**Affiliations:** 1 Department of Psychiatry, Rush University Medical Center, 1700 W. Van Buren St., Suite 5827A, Chicago, IL 60612 USA; 2 Institute of Child Development, University of Minnesota, Minneapolis, MN USA; 3 Department of Psychology, Emory University, Atlanta, GA USA

## Correction to: Child Psychiatry & Human Development 10.1007/s10578-018-0785-9

The article “Early Life Stress and Childhood Aggression: Mediating and Moderating Effects of Child Callousness and Stress Reactivity”, written by Dominika A. Winiarski, Melissa L. Engel, Niranjan S. Karnik and Patricia A. Brennan, was originally published electronically on the publisher’s internet portal (https://link.springer.com/article/10.1007/s10578-018-0785-9) on 13 February 2018 without open access.

With the author(s)’ decision to opt for Open Choice the copyright of the article changed on 18 February 2018 to © The Author(s) 2018 and the article is forthwith distributed under the terms of the Creative Commons Attribution 4.0 International License (http://creativecommons.org/licenses/by/4.0/), which permits use, duplication, adaptation, distribution and reproduction in any medium or format, as long as you give appropriate credit to the original author(s) and the source, provide a link to the Creative Commons license and indicate if changes were made.

The Original article has been corrected.

